# Minipuberty in Sons of Women with Low Vitamin D Status during Pregnancy

**DOI:** 10.3390/nu15224729

**Published:** 2023-11-09

**Authors:** Karolina Kowalcze, Robert Krysiak, Anna Obuchowicz

**Affiliations:** 1 Department of Pediatrics in Bytom, Faculty of Health Sciences in Katowice, Medical University of Silesia, Stefana Batorego 15, 41-902 Bytom, Poland; aobuchowicz@sum.edu.pl; 2 Department of Internal Medicine and Clinical Pharmacology, Medical University of Silesia, Medyków 18, 40-752 Katowice, Poland; rkrysiak@sum.edu.pl

**Keywords:** genital organs, hypothalamic–pituitary–gonadal axis, males, pregnancy complications, saliva, vitamin D status

## Abstract

Minipuberty is a transient phase of reproductive axis activation during the first several months of life, playing an important role in the development of reproductive organs in boys. Low 25-hydroxyvitamin D levels during pregnancy are associated with an increased risk of neonatal complications. An inadequate gestational vitamin D status is hypothesized to affect the postnatal activation of the hypothalamic–pituitary–gonadal axis. The purpose of our study was to assess whether a low vitamin D status during pregnancy determines the course of minipuberty in boys. The study included three groups of male infants born to women with different vitamin D statuses: sons of women with vitamin D deficiency (group 1), sons of women with vitamin D insufficiency (group 2), and male offspring of females with normal 25-hydroxyvitamin D levels (group 3 (the reference group)). Concentrations of testosterone, androstenedione, dehydroepiandrosterone sulfate, estradiol, progesterone, and 17-hydroxyprogesterone in saliva, as well as concentrations of gonadotropins in urine, were assayed monthly from postnatal months 1 to 6, and once every 2 months in the second half of the first year of life. Additionally, at each visit, penile length and testicular volume were assessed. Concentrations of testosterone, FSH, and LH, as well as penile length and testicular volume, were greater in group 1 than in groups 2 and 3. In turn, group 2 was characterized by higher FSH levels and a greater testicular volume than group 3. Peak concentrations of LH and testosterone were observed earlier in group 1 than in the remaining groups. The obtained results suggest that a low vitamin D status during pregnancy may have a stimulatory impact on reproductive axis activity and on the early postnatal development of male genital organs, correlating with the severity of hypovitaminosis D.

## 1. Introduction

Puberty, the process of physical maturation resulting in sexual maturity and attaining the capability of reproduction, is preceded by two earlier periods of activation of the reproductive axis. The first one takes place during fetal life, and determines prenatal genital development and dimorphic brain development [1]. The second period of the transient activation of the hypothalamic–pituitary–gonadal axis, known under the name of minipuberty, begins in the first postnatal week in response to the disappearance of placental hormones suppressing the secretion of gonadotropin-releasing hormone and gonadotropins: follicle-stimulating hormone (FSH) and luteinizing hormone (LH) [2,3,4]. In healthy male infants, the circulating levels of gonadotropins and testosterone gradually increase, reaching peak values between 1 and 3 months of life, and then drop to prepubertal levels between 6 and 9 months of age (FSH and LH), or at around 6 months of life (testosterone) [1,5]. Contrary to female infants, the peak LH levels in male infants during minipuberty are higher than FSH levels, which is accompanied by a higher testosterone concentration in boys than in girls [2]. The activation of the hypothalamic–pituitary–testicular axis is paralleled by an increase in the number of Leydig cells during the first three months of life, followed by the progressive apoptosis of fetal Leydig cells [6]. Despite a marked increase in testosterone concentration, its biological action in this period of life is limited by a simultaneous increase in the production of sex hormone-binding globulin and the lack of androgen receptors in some target cells (particularly in Sertoli cells) [4,7].

The physiological role of minipuberty is multidirectional, and still requires better understanding. In males, minipuberty promotes masculinization, including testicular and penile growth, and plays a role in the completion of testicular descent, if not already achieved at birth [4]. Penile growth velocity during the first 3 months of life (on average 1 mm per month) is considered to be an indirect marker of testosterone levels during this period of life [8]. Some authors have observed an increase in testicular volume, not detectable by palpation, in the first 5–6 months of life, which, after completing minipuberty, was followed by a decrease in testicular volume [9]. The activation of the hypothalamic–pituitary–gonadal axis is implicated in the proliferation of Sertoli cells in the seminiferous tubules and the differentiation of gonocytes into adult dark spermatogonia, which is important for later fertility potential [2]. Lastly, differences in sex hormones during minipuberty determine sexual dimorphism in linear growth and body composition, and are responsible for a higher growth velocity and the accumulation of more lean mass in males compared to females [6,7].

The results of numerous studies have indicated that vitamin D (calciferol) plays a relevant role in the development and functioning of the reproductive system. A meta-analysis of six studies, including 3016 precocious puberty patients and 8296 healthy individuals, showed that vitamin-D-deficient children were more likely to develop precocious puberty than vitamin-D-sufficient children [10]. Another meta-analysis showed that 25-hydroxyvitamin D levels were lower in individuals with idiopathic central puberty than in healthy individuals [11]. Low 25-hydroxyvitamin D levels were also observed in variants of precocious puberty: premature adrenarche [12] and premature menarche [13]. Lastly, first pregnancy trimester between November and April, a period of the year when the sunlight-induced skin synthesis of endogenous calciferol is decreased [14], was associated with earlier pubertal timing both in girls and boys, which was attributed to lower 25-hydroxyvitamin D levels [15]. Vitamin D receptors and enzyme-metabolizing calciferol are expressed in Sertoli cells, Leydig cells, germ cells, spermatozoa, and in the epithelial cells lining the male reproductive tract [16]. Lastly, vitamin-D-deficient men and mice lacking the vitamin D receptor are characterized by decreased sperm counts and a reduced sperm motility [17].

A low vitamin D status during pregnancy is associated with adverse neonatal pregnancy outcomes, including an increased risk of preterm birth, low birth weight, and giving birth to a child small for gestational age [18,19]. Risk of all these complications was found to correlate with the severity of vitamin D deficiency, while achieving optimal levels of 25-hydroxyvitamin D through vitamin D supplementation reduced the prevalence of these complications [20]. Interestingly, both prematurity and a birth weight of less than the 10th percentile for gestational age, falling within the first weight decile, or below a −2 standard deviation were found to modulate the course of minipuberty in boys [21,22,23,24]. The circulating levels of testosterone and LH were higher in premature than full-term boys, which was accompanied by faster testicular and penile growth in the former group of patients [21]. Boys born small for gestational age were characterized by higher LH levels [22], higher [23] or lower [22] FSH concentrations, and higher [24] or lower [22] testosterone concentrations than boys born appropriate for gestational age. In turn, postpubertal boys born small for gestational age had a reduced testicular volume, increased levels of LH, and lower levels of testosterone than their peers born appropriate for gestational age [25]. Lastly, low maternal 25-hydroxyvitamin D levels during pregnancy were associated with worse neurocognitive development [26].

To date, only one previous study has focused on investigating the relationship between vitamin D status and minipuberty. Kılınç et al. [27] observed that girls aged 30–45 days with serum 25-hydroxyvitamin D concentrations above 20 ng/mL had lower circulating total testosterone levels than girls with 25-hydroxyvitamin D levels below 10 ng/mL or between 10 and 20 ng/mL. In turn, inhibin B concentrations were lower in girls with 25-hydroxyvitamin D levels below 10 ng/mL than those with 25-hydroxyvitamin D levels between 10 and 20 ng/mL or above 20 ng/mL. Unlike female infants, the authors observed no differences in the hormone levels in infant boys. Unfortunately, hormone levels were assessed only in one time point, serum total testosterone was the only measured androgen, the authors did not provide information concerning maternal health during pregnancy, and they did not assess any clinical outcomes. The important physiological role of minipuberty, the paucity of data, and the limitations of available studies encouraged us to evaluate whether a low vitamin D status during pregnancy has a modulatory impact (stimulatory or inhibitory) on male minipuberty and the development of genital organs.

## 2. Materials and Methods

The study protocol was reviewed and approved in advance by the local ethics committee. Parents provided written informed consent on behalf of the participating infants after receiving a full explanation about the study, its purpose, and potential risks. All procedures were conducted in accordance with the 1964 Declaration of Helsinki and its subsequent revisions. As our study was an observational study, it did not need to be registered in a public database. The findings were analyzed and reported in accordance with STROBE (Strengthening the Reporting of Observational Studies in Epidemiology) guidelines.

### 2.1. Participants

This single-center, matched cohort study included a group of 96 apparently healthy boys in the first month of life. The study population was ethnically homogeneous (all participants and their mothers were white Polish Caucasians). The Upper Silesia, the area where the study was carried out, is an urban area in southern Poland. In line with recommendations on vitamin D prophylaxis, since birth, all the included infants received exogenous vitamin D (10 µg (400 U) daily) [28]. Based on the vitamin D status of their mothers during pregnancy, they were classified into one of three groups. Group 1 included sons of women with vitamin D deficiency, defined as plasma 25-hydroxyvitamin D levels below 50 nmol/L (20 ng/mL). Group 2 enrolled male offspring of women with vitamin D insufficiency, defined as circulating 25-hydroxyvitamin D concentrations between 50 and 75 nmol/L (20 and 30 ng/mL). In turn, group 3 (the reference group) included sons of women with a normal vitamin D status, which was diagnosed if plasma 25-hydroxyvitamin D levels ranged between 75 and 150 nmol/L (30 and 60 ng/mL) [29]. The study only included the offspring of women in whom at least two measurements in two different trimesters of the same pregnancy provided consistent results. The sons of women with only one abnormal result, those with discordant findings during pregnancy (e.g., one result suggesting vitamin D deficiency and another one suggesting vitamin D insufficiency), and those with 25-hydroxyvitamin D concentrations above 150 nmol/L (60 ng/mL) were not considered eligible for the study. The study population was selected from a larger cohort of boys meeting the inclusion criteria (n = 182), in order to create three study groups matched for maternal age, education, occupational activity, gestational age of delivery, and the number of deliveries. The algorithm used in the matching procedure was based on the minimum Euclidean distance rule. Because of anticipated dropouts and noncompliance, the number of participants in each group (n = 32) exceeded the calculated sample size. A calculation performed before conducting the study showed that at least 26 patients per group were required to detect a 20% between-group difference in saliva testosterone levels (the primary endpoint) with an 80% power, given a type I error probability of 0.05. The expected probability of the primary endpoint was estimated based on our pilot findings. In order to limit the influence of seasonal confounds and fluctuations in the concentrations of 25-hydroxyvitamin D and the remaining variables, the study included the same (n = 24) numbers of infants conceived in spring (8 in group 1, 7 in group 2, and 9 in group 3), summer (8 in each group), autumn (7 in group 1, 9 in group 2, and 8 in group 3), and winter (9 in group 1, 8 in group 2, and 7 in group 3).

The exclusion criteria included: major congenital anomalies, genetic syndromes, congenital infections, metabolic disorders, delivery before week 36 of gestation, unilateral or bilateral cryptorchidism, in utero alcohol exposure, birth asphyxia, and chronic pharmacotherapy. Potential participants were also excluded if their mothers had genetic syndromes or chronic disorders diagnosed before pregnancy, during pregnancy, or postpartum, developed acute complications requiring hospitalization during pregnancy, or received any treatment for a period longer than seven days (except for micronutrient and vitamin preparations for pregnant women).

### 2.2. Study Design

**Figure 1:**
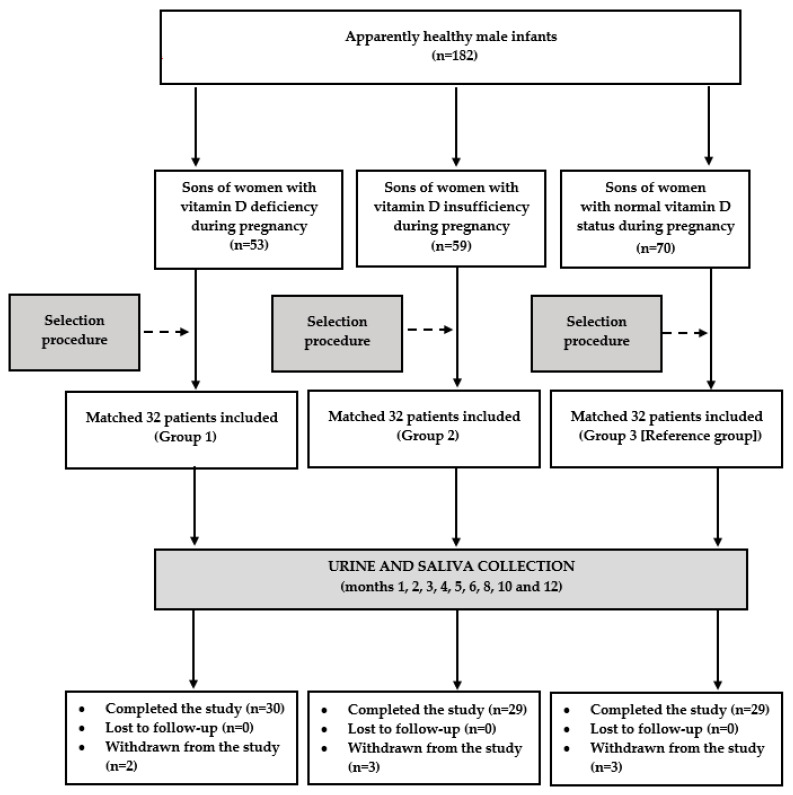
Flow of the patients through the study.

The flow of patients through the study is depicted in Figure 1. In order to perform anthropometric assessments of the patients, to reduce the risk of preanalytical errors associated with the collection, contamination, and transport of the urine and saliva samples, and to improve patient safety, the children accompanied their parents during each study visit. The participants were followed-up until the end of the first year of life: once a month from month 1 to 6, and once every 2 months thereafter. Over the entire study period, all the participants continued vitamin D supplementation at the same dose as before. During each visit, the parents were asked to assess their child’s health since the last visit. They were interviewed about progress in reaching age-appropriate developmental milestones, outpatient or emergency department visits, hospitalizations, treatments, vaccinations, injuries, and concerns the parent had about the child’s development or behavior. Moreover, the investigator carried out a detailed physical examination of the infant, and analyzed the results of performed laboratory tests. The information obtained from the parents, the results of the physical examination, and the results of laboratory tests (if performed) enabled the investigators to determine the child’s health status. Urine and saliva samples were collected if the infant was healthy and had not been treated with any medications (except for vitamin D and mandatory vaccines) in the last 10 days. The infants’ results were statistically analyzed if the biological material for the laboratory tests was obtained from the patient on seven or more occasions (at least five times in the first six months of life).

The anthropometric evaluation included an assessment of the infants’ length, weight, body mass, and head circumference. Recumbent length was measured using an infantometer from the top of the head to the soles of feet to the nearest 0.1 cm (Seca, Hamburg, Germany). Weight was measured with an electronic scale accurate to 0.1 kg (Seca 834, Hamburg, Germany). The body mass index was calculated by dividing the weight by the length squared. The head circumference was measured to the nearest 0.1 cm with a non-stretchable tape placed anteriorly on the forehead just above the eyebrows and posteriorly at the maximum protrusion of the occiput.

The penile length was assessed in the supine position with flexed legs using a calibrated tape. The measurements were performed twice by the same investigator (Karolina Kowalcze) in order to avoid any interobserver differences that may arise, and the mean of the two measurements was used for the analysis. The penile length was measured from the symphysis pubis to the tip of phallus glans in complete stretching. The length of the foreskin was not included in the measurement.

After having measured the penile length, the principal investigator (Karolina Kowalcze) performed a testicular ultrasound using a high-frequency (5–12 MHz) linear-array transducer (Esaote MyLab Six, Genoa, Italy). The size of the left testis was measured first, followed by the right testis. Three separate transverse and sagittal images were recorded for each testis. After the length, width, and height were obtained in the ultrasound, the testicular volume was calculated using the empiric formula: length × width × height × 0.71. The epididymis was not included in the volume measurement. The volumes of both testes calculated based on each set of data were then averaged. The mean testicular volume was calculated as the sum of the right and left testes’ volumes divided by two.

### 2.3. Laboratory Assays

Urine and saliva samples for the laboratory analysis were collected between 7.00 and 8.30 a.m. in a quiet and air-conditioned room (constant temperature of 23–24 °C). Urine samples were collected using a sterile pediatric urine collection bag (Medicavera, Szczecin, Poland). After cleansing the infant’s genital area and allowing the newly cleansed area to air dry, the pediatric urine collection bag was firmly attached to the child’s genital area. Immediately after the infant voided into the collection bag, the bag was removed from the infant, and, after cutting its corner with scissors, the urine was poured into the sterile container. Saliva samples were collected by the main researcher (Karolina Kowalcze) via careful aspiration with the use of 2 mL sterile syringes. The blunt tips of the syringes were positioned in the oral cavity for 30–50 s, and saliva samples were obtained using a low-pressure suction. Infants were not fed for at least 1 h prior to the procedure in order to limit the impact of food contamination and to exclude a possible impact of feeding on secretion and/or metabolism of the assessed hormones. The extracted saliva samples were centrifugated, and the supernatants were aliquoted to storage vials and frozen. The samples of urine and saliva supernatants were stored at −20 °C, and thawed immediately prior to analysis. All measurements were performed in duplicate in order to smooth possible intra-sample variance by a person blinded to all clinical and diagnostic information. Urine gonadotropins were determined using a solid-phase, two-site chemiluminescent immunometric assay (Siemens Healthcare Diagnostics, Erlangen, Germany). The FSH and LH levels in the urine were then divided by the creatinine concentrations to correct for differences in water excretion. Urine creatinine was measured using a routine method (Roche Diagnostics, Basel, Switzerland). The concentrations of testosterone, androstenedione, dehydroepiandrosterone sulfate (DHEA-S), estradiol, progesterone, and 17-hydroxyprogesterone in the saliva were measured using an enzyme-linked immuno-sorbent assay with reagents obtained from Diametra (Perugia, Italy), BioVendor R&D (Brno, Czech Republic) and IBL International (Hamburg, Germany). The limits of detection (LOD) are listed in Table 1.

### 2.4. Covariates

Potential confounding factors were identified based on the existing literature and clinical rationale. Information on the maternal age at delivery, the number of deliveries, gestational age of delivery, birth order, smoking, mean body mass index during pregnancy, and mean blood pressure during pregnancy was extracted from the mothers’ and infants’ medical records stored online in a digital format. The necessary data concerning education, occupational activity, a type of work, and breastfeeding were obtained by interviewing the mothers during the first visit. The daily and cumulative vitamin D intakes were calculated based on an analysis of individual dietary questionnaires completed by the mothers during their pregnancy (calciferol contained in food) and based on an analysis of the mothers’ medical records (calciferol contained in supplements for pregnant women). The total vitamin D intake by the participants (including the cholesterol present in breast milk/milk formula and in supplements for infants) was calculated based on information provided by the parents and contained in the child’s health booklet. The infant’s length, weight, body mass index, and head circumference were obtained via anthropometric measurements. Because all the participants and their mothers were white Polish Caucasians, ethnicity was controlled for by design. Owing to the sample size, matching the groups for hard variables, no between-group differences in covariates at baseline, a risk of making type I error associated with over-stratification, and a possible impact of interobserver reliability on some covariates, the results were not adjusted for confounding factors.

### 2.5. Statistical Analysis

All the statistical analyses were performed using the Statistica 12.0 PL software (StatSoft Polska, Kraków, Poland). Before entering the statistical analysis, all the outcome variables were log transformed in order to accomplish the assumptions of normality. Quantitative data at different time points were compared using a repeated-measures analysis of variance test, followed by Tukey’s post hoc test for pairwise comparisons. Comparisons between the groups at the same time points were carried out using a one-way analysis of variance followed by Bonferroni’s test. Categorical data were analyzed using the chi square test. Relationships between the outcome measures were quantified using Pearson’s r tests (for two continuous variables), phi coefficient (for one continuous and one categorical variable), and point-biserial (for two categorical variables). Moreover, in order to increase the power and investigate a potential dose dependency, a linear regression analysis was carried out with maternal 25-hydroxyvitamin D concentrations as an independent continuous variable, and testosterone concentrations, FSH concentrations, LH concentrations, penile length, and testicular volume as dependent variables. Data with *p*-values corrected for multiple comparisons of below 0.05 were considered to be significantly different.

## 3. Results

There were eight withdrawals during the study. Repeated infections of the upper respiratory tract in three infants (one from group 1 and two from group 3) made it impossible to collect the required number of urine and saliva samples. Two infants (assigned to groups 2 and 3) did not complete the study because they needed chronic treatment of recurrent convulsions and of gastroesophageal reflux. Two infants assigned to group 2 stopped participating in the study due to a change of residence address. Lastly, the mother of one boy (from group 1) got pregnant again, and found further regular visits to be inconvenient and impossible. Eighty-eight infants (92%) completed the study, and only their results were analyzed. A post hoc power analysis showed that the study had sufficient power (83%) to detect assumed differences in the primary endpoint.

There were no between-group differences in age, education, occupational activity, type of work, the number of deliveries, smoking, body mass index, and blood pressure (both systolic and diastolic) between the mothers of the participating infants. The mean circulating 25-hydroxyvitamin D levels, daily vitamin D intake, and cumulative vitamin D intake in pregnancy were the highest in group 3 and lowest in group 1 (Table 2).

There were no differences between the sons of mothers with vitamin D deficiency, vitamin D insufficiency, and a normal vitamin D status in gestational age of delivery, birth order, length, weight, body mass index, head circumference, breastfeeding, and total daily (with food and supplementation) vitamin D intake (Table 3). At the beginning of the study, there were no infants with hormone levels below the LOD. Throughout the study, vitamin D intake did not differ between the groups.

In all the study groups, testosterone was detectable in the saliva during the first six months of life. In group 1, the highest concentrations of testosterone were observed in month 1, while in the remaining two groups in month 2. During the first five months of life, the salivary testosterone concentrations were higher in group 1 than in the remaining groups (Table 4).

In all the study groups, androstenedione was detectable in the saliva from month 1 to month 6. The androstenedione levels were at a similar level between months 1 and 4, and decreased thereafter. There were no differences in the salivary levels of this hormone between patients with various vitamin D statuses (Table 5).

Detectable salivary DHEA-S concentrations were observed over the entire study period. There were no differences in the DHEA-S levels between patients with different vitamin D statuses and between different time points in the same group (Table 6).

Detectable salivary estradiol concentrations were observed from month 1 to month 3. These concentrations did not differ between the study groups. In each study group, the estradiol concentrations were similar at different time points (Table 7).

The presence of progesterone and 17-hydroxyprogesterone in the saliva was observed over the entire study period. However, there were no differences in these hormones between the study groups and between different time points in the same group (Table 8 and Table 9).

The presence of FSH in the urine was observed during the first eight months of life. From month 1 to 6, the FSH concentrations were higher in group 1 than in the remaining two groups, while, during the first five months of life, these concentrations were higher in group 2 than group 3 (Table 10).

In all the study groups, LH was detectable in the saliva during the first six months of life. Peak concentrations of this hormone in group 1 were observed in the first month of life, while in the remaining groups, in the second month of life. Between month 1 and month 6, the urine LH levels were higher in group 1 than in the remaining groups (Table 11).

During the study, penile length increased with time. From postnatal month 4, the length was greater in group 1 than in the remaining two groups. No differences were observed between groups 2 and 3 (Table 12).

In all the study groups, testicular volume increased during the first five months of life. No significant changes were observed thereafter. From month 3, the testicular volume was greater in group 1 than in the remaining groups, while, from month 5, the value of this parameter was greater in group 2 than group 3 (Table 13).

From month 1 to month 6, salivary testosterone concentrations were positively correlated with urinary LH levels (group 1: r values between 0.46 (*p* = 0.0001) and 0.60 (*p* < 0.0001) depending on time point; group 2: r values between 0.52 (*p* < 0.0001) and 0.65 (*p* < 0.0001) depending on time point; and group 3: 0.50 (*p* < 0.0001) and 0.67 (*p* < 0.0001) depending on time point). There were also positive correlations between testosterone levels and penile length (group 1: r values between 0.31 (*p* = 0.0204) and 0.40 (*p* = 0.0008) depending on time point; group 2: r values between 0.34 (*p* = 0.0218) and 0.46 (*p* = 0.0002) depending on time point; and group 3: r values between 0.35 (*p* = 0.0104) and 0.43 (*p* = 0.0008) depending on time point). Moreover, FSH was positively correlated with testicular volume (group 1: r values between 0.29 (*p* = 0.0408) and 0.35 (*p* = 0.0138) depending on time point; group 2: r values between 0.36 (*p* = 0.00155) and 0.43 (*p* = 0.0006) depending on time point; and group 3: r values between 0.43 (*p* = 0.0005) and 0.49 (*p* = 0.0001) depending on time point). In group 1, 25-hydroxyvitamin D levels were inversely correlated with testosterone concentrations (r values between −0.35 (*p* = 0.0198) and −0.43 (*p* = 0.0006) depending on time point), LH levels (r values between −0.32 (*p* = 0.0208) and −0.40 (*p* = 0.0008) depending on time point), and FSH levels (r values between −0.42 (*p* = 0.0005) and −0.49 (*p* = 0.0001) depending on time point). In group 2, 25-hydroxyvitamin D levels were inversely correlated with FSH levels (r values between −0.40 (*p* = 0.0008) and −0.46 (*p* = 0.0002) depending on time point. Lastly, there were correlations between maternal 25-hydroxyvitamin D levels and vitamin D intake (r values between 0.67 (*p* < 0.00001) and 0.74 (*p* < 0.0001) depending on the study group). The salivary and urinary levels of the investigated hormones, penile length, and testicular volume did not correlate with the daily and cumulative vitamin D intake by the pregnant women and with the intake of this vitamin by the participants.

In the linear regression analysis, maternal 25-hydroxyvitamin D levels showed a relationship with FSH levels, LH levels, testosterone levels, penile length, and testicular volume (Table 14). The dose–response analysis showed that each 10 nmol/L decrement in 25-hydroxyvitamin D concentrations was associated with a statistically significant (*p* < 0.05) increase in all these variables, the degree of which depended on the time point: 7–10% for testosterone, 14–16% for FSH, 9–11% for LH, 1–3% for penile length, and 2–4% for testicular volume.

## 4. Discussion

The most important finding of the current study was that vitamin D deficiency in women during pregnancy impacted the course of minipuberty in their male offspring by affecting pituitary gonadotropin secretion, testicular testosterone production, and the growth of male genital organs. The effect on minipuberty was more pronounced in the sons of women with vitamin D deficiency than insufficiency. These findings, the results of the regression analysis, as well as the inverse correlations between maternal 25-hydroxyvitamin D levels during pregnancy and the concentrations of gonadotropins and testosterone in the infants, suggest that alterations in the postnatal activity of the infant’s reproductive axis and the accelerated growth of genital organs are most pronounced in descendants of mothers with the lowest maternal 25-hydroxyvitamin D levels. Strict inclusion criteria and numerous exclusion criteria minimized a possible impact of concurrent disorders or concomitant therapies in both the mothers and their descendants. Unfortunately, no study evaluated the course of minipuberty in children born to women with chronic disorders, while, in the affected infants, this assessment was limited to children born prematurely, small for gestational age, or with genetic syndromes (Klinefelter syndrome, Turner syndrome, or Prader–Willi syndrome) [5]. This makes it difficult to conclude whether our findings are a unique consequence of an impaired action of calcitriol (and possibly also of other vitamin D metabolites) or represent a non-specific response to the impairment of fetal homeostasis during pregnancy. Minipuberty is preceded by the intrauterine activation of the reproductive axis between gestational weeks 10 and 24 [1]. Interestingly, most measurements of 25-hydroxyvitamin D in the mothers of the boys participating in our study were made in the first two trimesters of pregnancy. This similarity suggests that the intrauterine phase of maturation may have an impact on the course of the subsequent postnatal phase. Alternatively, alterations in minipuberty in the sons of women with a low calciferol status in pregnancy may result from chronic changes in the intrauterine environment, which were found to affect fetal programming [30] and may, in this way, influence the second phase of the activation of the reproductive axis.

Interestingly, peak concentrations of LH and testosterone were observed earlier in the sons of women with vitamin D deficiency (month 1) than in the other boys participating in the study (month 2). This finding suggests that vitamin D deficiency is associated with an earlier activation of the reproductive axis after birth. It is possible that a low calciferol status during pregnancy partially explains why the urinary LH and testosterone levels in the study by Kuiri-Hänninen et al. [21], as well the testosterone concentrations in the saliva and serum in the study by Contreras et al. [31], had already reached their peak values at month 1. Although both research teams did not assess vitamin D status, calciferol deficiency is frequently diagnosed, while mean 25-hydroxyvitamin D levels are low in the general populations of pregnant Finnish [32] and American [33] women. Certainly, we cannot exclude the impact of other factors contributing to differences in the course of minipuberty between the boys participating in these studies and those in our study. Due to numerous exclusion criteria, our study population was probably healthier in comparison to the boys participating in the study by Kuiri-Hänninen et al. [21]. Moreover, the current study was the only one excluding the descendants of women with comorbidities or receiving any chronic treatment (treated longer than for 7 days). Lastly, the observed differences may be associated with differences in the biological material and/or in assay methods.

Testosterone was the only androgen, the salivary levels of which differed between patients with different calciferol statuses. As in the case of testosterone, androstenedione was detectable in the saliva for the first six months of life, while detectable DHEA-S levels were observed over the entire observation period. Although the salivary DHEA-S concentrations were higher than the concentrations of the remaining steroid hormones, they were much lower in comparison to the plasma or serum of prepubertal children [34]. This finding may be explained by its biochemical properties. DHEA-S cannot penetrate through cell membranes into the saliva, and small salivary levels of this steroid are a consequence of the paracellular transport through the tight junctions lining the salivary acini and the lumen of the salivary glands [35]. The lack of between-group differences in androstenedione and DHEA-S levels, as well as in progesterone and 17-hydroxyprogesterone, and the stable concentrations of all these hormones over the entire study period suggest unaltered activity of the enzymes determining the synthesis of testosterone upstream hormones: 20,22-desmolase, 17α-hydroxylase/17,20-lyase, steroid sulfatase, steroid sulfotransferase, and 3β-hydroxysteroid dehydrogenase [36] during the first year of life, and their resistance to changes in calciferol content. Although it was not the aim of our research, the obtained results suggest that an assessment of the androgen concentrations in the saliva may be helpful in the diagnosis and follow-up of boys with disorders of androgen production. Salivary measurements may be particularly suitable for boys with congenital adrenal hyperplasia, one of the most common hereditary diseases [37], if not diagnosed by neonatal screening (the non-classic form of this disorder or, rarely, the simple virilizing form) or in order to monitor glucocorticoid treatment. As our study shows, saliva collection is simple, non-invasive, painless, low in stress, and well tolerated by infants. For all these reasons, measurements of the steroid hormones in the saliva may be repeated even many times, without posing a risk to children.

We can only hypothesize how an abnormally low vitamin D status in pregnancy modulates the course of minipuberty in the offspring. An increased production of both gonadotropins and testosterone, positive correlations between LH and testosterone levels, and the similar strength of these correlations suggest the activation of the reproductive action at the level of the hypothalamus and/or pituitary rather than a dominant direct effect on Leydig cells. Some indirect pieces of evidence are in line with this interpretation. The testicular production of testosterone from androstenedione is catalyzed by 17β-hydroxysteroid dehydrogenase type 3 [36]. Although no study has assessed the relationship between calciferol or its metabolites and the activity of this isoenzyme, calcitriol was found to upregulate 17β-hydroxysteroid dehydrogenase types 2, 4, and 5 in prostate cancer cells (but not in stromal cells) [38]. Moreover, vitamin D receptor knock-down Leydig cells were characterized by down-regulated testosterone synthesis [39]. These findings suggest that low vitamin D acting directly at the testicular level should rather reduce testosterone synthesis. In turn, the opposite effect, which is line with our findings, was found to be exerted at the level of pituitary cells. A low calciferol status attenuated the inhibitory effect of metformin on the gonadotropin levels observed in vitamin-D-sufficient women [40]. Moreover, the degree of reduction in gonadotropin levels in this study was positively correlated with 25-hydroxyvitamin D levels [40]. This effect may be mediated by 5′-adenosine monophosphate-activated protein kinase, a key sensor of energy homeostasis [41]. Gonadotropin-producing cells (gonadotropes) are the pituitary cells with the highest expression of this enzyme [42]. Moreover, an insufficient calciferol supplementation was found to down-regulate the 5′-adenosine monophosphate-activated protein kinase pathway [43], and its increased activity in the pituitary was found to be associated with decreased FSH and LH secretion in response to gonadotropin secretagogues [42]. Lastly, the 5′-adenosine monophosphate-activated protein kinase pathway seems to play an important role in the regulation of fetal programming [44], as well as in the regulation of gonadotrope secretory function and testicular steroidogenesis during fetal life [45].

It is worth underlying that the outcome variables did not correlate with maternal calciferol intake (both daily and cumulative), despite the between-group differences in vitamin D consumption and the presence of strong correlations between calciferol intake and 25-hydroxyvitamin D levels in the mothers. These findings argue that changes in the course of minipuberty reflect long-term vitamin D status in pregnancy, and do not seem to result from a direct pharmacokinetic effect of exogenous vitamin D on the reproductive axis and genital organs. They may serve as an argument in favor of the routine assessment of 25-hydroxyvitamin D, at least in some groups of apparently healthy pregnant women (particularly pregnant women with a history of premature delivery or giving birth to a child small for gestational age during previous pregnancy). Our findings cannot also be explained by abnormalities in vitamin D homeostasis in postnatal life, because, over the entire first year of life, both before and during the study, the children received the recommended dose (10 µg (400 U) daily) of exogenous calciferol [28], there were no differences in total vitamin D intake in the infants, and the outcome measures did not correlate with vitamin D intake.

The obtained results support previous observations linking minipuberty in boys with the growth of genital organs in the first months of life [8,9,21]. Maternal hypovitaminosis D during pregnancy seems to have an impact on penile and testicular enlargement in infancy. Positive correlations with testosterone, but not with DHEA-S and androstenedione, indicate that penile enlargement during minipuberty is stimulated by testosterone, and possibly also by downstream androgens (particularly dihydrotestosterone), but not by the upstream steroid hormones assessed in the current study. Intergroup differences in testosterone may explain why, from the fourth month of life, the sons of vitamin-D-deficient mothers were characterized by a greater penile length in comparison to the remaining two groups. Interestingly, a similar strength of correlations between salivary testosterone and penile length in all the study groups suggests that the impact of calciferol and/or its metabolites on this parameter is associated mainly or exclusively with the activation of the hypothalamic–pituitary–gonadal axis. In turn, testicular enlargement, most pronounced in the sons of vitamin-D-deficient mothers and least pronounced in the descendants of mothers with a normal vitamin D status, seems to be only in part secondary to an increased secretion of FSH, a hormone playing a fundamental role in the proper functioning of Sertoli cells [46]. Unlike the correlations with penile length, the study groups differed in the strength of the correlations between FSH and testicular volume, which were weakest in the sons of mothers with the lowest 25-hydroxyvitamin D levels. This observation suggests that the role of FSH as a stimulator of testicular growth is less pronounced if calciferol availability in pregnancy is reduced. In sons are born to mothers with a low calciferol status, pituitary-independent mechanisms are implicated to a greater degree in the modulation of Sertoli and/or germ cell function than in male infants born to mothers with a normal vitamin D status. These mechanisms are not mediated or modulated by altered testosterone production because of the lack of androgen receptors in Sertoli cells during infancy [5], which is supported by the lack of correlation between the salivary testosterone and testicular volume in our study. More likely is an association with the pleiotropic effects of calciferol, which was found to exert anti-inflammatory effects, improve endothelial function, increase insulin sensitivity, reduce oxidative stress, and have neuroprotective properties [47,48]. Thus, a decreased vitamin D supply may impair the functioning of Sertoli cells (and possibly also of early germ cells) through an impact on the autocrine or paracrine regulators of these cells, which are more sensitive to changes in their microenvironment than Leydig cells are [49].

Another interesting finding of our study was differences in the FSH levels and testicular volume between the male offspring of women with vitamin D deficiency and vitamin D insufficiency. Moreover, unlike LH and testosterone, both the FSH concentrations and testicular volume were greater in the infant boys born to mothers with mild disturbances in vitamin D homeostasis during pregnancy in comparison to the sons of women with a normal calciferol status. Our results do not support those obtained by another research group. Gaml-Sørensen et al. recently reported that the adult sons of mothers with 25-hydroxyvitamin D levels below 75 nmol/L had a lower average testicular volume compared to the sons of mothers with 25-hydroxyvitamin D levels above 75 nmol/L, and that the risk of a low testicular volume was highest if the maternal 25-hydroxyvitamin D concentrations during pregnancy were below 25 nmol/L [50]. Thus, it is possible that more pronounced testicular enlargement during minipuberty in the descendants of women with an inadequate gestational vitamin D status may have an unfavorable impact on testicular growth during the later stages of sexual maturation. Our findings may also suggest that the early stages of spermatogenesis are more susceptible to small differences in vitamin D homeostasis in comparison to testicular steroidogenesis, which may be explained by the putative action of active vitamin D at the level of both gonadotropes and Sertoli cells. In line with this explanation, the expression of the vitamin D receptor was reported to be markedly higher in Sertoli cells when compared to Leydig cells [51,52]. Moreover, correlations between FSH and testicular volume in our study differed between the groups, depending on the maternal 25-hydroxyvitamin D levels. Thus, our observations are in favor of assuming 75 nmol/L as a threshold value for vitamin D concentration during pregnancy. To better understand the role of Sertoli cells in the development of minipuberty complications secondary to disturbances in vitamin D homeostasis, it would be helpful to evaluate markers of Sertoli cell health and number: anti-Müllerian hormone and inhibin B [53]. Unfortunately, contrary to some proteins (e.g., insulin) actively transferred from the place of synthesis into the salivary glands and secreted into the saliva through exocytosis [54], no similar data are available for both Sertoli cell products. In turn, frequent blood collections from otherwise healthy small children were unacceptable from an ethical point of view. Lastly, poor knowledge of the pharmacokinetic properties of anti-Müllerian hormone and inhibin B makes it impossible to assess the circulating pools of both hormones on the basis of their urine excretion. Presently, we are conducting a study aimed at addressing this question in adult volunteers. In the case of encouraging results, we intend to evaluate the urine levels of both hormones in minipubertal children to assess Sertoli cell function in this period of life.

Our study also shows that saliva contains detectable concentrations of estradiol. A transient increase in the circulating levels of this hormone is a typical feature of minipuberty in females {1, 5]. However, the estradiol levels in girls fluctuate, which can be explained by alternating periods of maturation and atrophy of the ovarian follicles, and the decline in estradiol levels is observed only in the second year of life [5]. Our findings indicate that male minipuberty is characterized by the short-term (during the first three months of life) presence of this hormone in the saliva, which, during this time, remains at a similar level. Intergender differences in minipubertal estradiol in comparison to previous studies may reflect much lower circulating concentrations of this hormone in males than females during infancy [55], probably resulting from sex-dependent differences in estrogen secretion [56]. The assessment of estrogen production by the endocrine organs of children who died suddenly during their first two years of life showed that the ovaries contained larger amounts of estrogens than the testes did, though adrenal estradiol (and estrone) content did not differ between male and female infants [56]. Differences between estradiol production may additionally be associated with very low activity of aromatase in the peripheral tissues of boys before puberty [57]. Despite detectable levels, we did not observe differences in salivary estradiol levels and their dynamics between the infants of women with different vitamin D statuses. This finding is in disagreement with a well-documented association between hypovitaminosis D and the earlier development and faster progression of puberty in girls [10,11]. It is difficult to determine the practical significance of this finding. In neither study group did estradiol concentrations correlate with gonadotropin and testosterone levels, or with penile length and testicular volume. Thus, estradiol in minipubertal boys, unlike adult men [58], is probably not implicated in mediating the testosterone action on gonadotropin production. Moreover, contrary to testosterone, estradiol is unlikely to play a role in the development of male genital organs, at least during the first several months of life.

There are some methodological limitations to the study that need to be taken into account. The relatively small number of participants limits the extent of the conclusions that can be drawn. Cohort and single-center studies are prone to bias and confounding, which may affect the validity and reliability of the obtained results. Because the recruitment took place several days after the birth, the study design did not allow for the detection of between-group differences in the investigated parameters in the first hours and days of life. An adjustment for prognostic covariates, not performed in the current study, would help to explain some of the variation in the outcome variables between the patients. Although immunoassays are frequently used in laboratory medicine for the quantitation of the circulating levels of steroid hormones, mass-spectrometry-based methods are recognized as a gold standard tool for the determination of these hormones [59]. Although the inclusion of only apparently healthy boys enabled us to create a homogenous group of participants, the lack of girls and children with comorbidities did not allow us to conclude about hormonal and clinical changes related to minipuberty in these groups. Altered minipuberty in the descendants of women with vitamin D excess, not participating in the study, cannot be excluded. Lastly, the study does explain the cellular and molecular mechanisms’ underlying differences in the course of minipuberty between sons of mothers with different vitamin D statuses.

## 5. Conclusions

Compared to the male descendants of women with 25-hydroxyvitamin D concentrations within the reference range, infant males born to women with vitamin D deficiency had higher levels of testosterone and gonadotropins. The male offspring of vitamin-D-deficient women were also characterized by earlier peak activity of the hypothalamic–pituitary–testicular axis. These hormonal differences may contribute to faster penile growth and a larger testicular volume in the first several months of life in the sons of vitamin-D-deficient women. Maternal vitamin D insufficiency was associated with increased FSH levels and testicular volume, suggesting discrete abnormalities in the functioning of Sertoli cells. The obtained results suggest that a low calciferol status impacts the course of minipuberty in boys, and the strength of this effect depends on the severity of hypovitaminosis D. Considering the putative role of minipuberty in the development of reproductive health, our findings argue that even mild disturbances of calciferol homeostasis in pregnancy should be diagnosed and effectively treated. Due to a small number of participating boys and methodological limitations, as well as because of the lack of similar studies, the obtained results should be interpreted with caution, and verified in larger, well-designed clinical studies. They should be also an incentive to analyze the impact of other micronutrient and macronutrient deficiencies and their supplementation on the hormonal and clinical aspects of minipuberty.

## Figures and Tables

**Table 1:** Limits of detection of steroid hormones and gonadotropins.

Hormone	Value
Salivary testosterone	10 pmol/L
Salivary androstendione	18 pmol/L
Salivary DHEA-S	100 nmol/L
Salivary estradiol	4 pmol/L
Salivary progesterone	16 pmol/L
Salivary 17-hydroxyprogesterone	11 pmol/L
Urinary FSH	0.1 U/L
Urinary LH	0.1 U/L

Abbreviations: DHEA-S—dehydroepiandrosterone sulfate; FSH—follicle-stimulating hormone; and LH—luteinizing hormone.

**Table 2:** Characteristics of mothers of the participating infants.

Variable	Group 1	Group 2	Group 3
Number (n)	30	29	29
Age (years)	31 ± 8	30 ± 7	32 ± 8
Primary or vocational/secondary/university education (%)	30/30/40	31/34/34	28/34/38
Occupational activity/white-collar/pink-collar/blue-collar workers (%)	80/27/27/27	83/28/24/31	83/31/21/31
Number of deliveries (n)	1.7 ± 0.7	1.5 ± 0.7	1.7 ± 0.7
Smokers (%)	27	24	28
Body mass index (kg/m^2^)	24.3 ± 4.0	23.9 ± 3.5	23.6 ± 3.7
Systolic blood pressure (mmHg)	122 ± 17	119 ± 18	117 ± 19
Diastolic blood pressure (mmHg)	80 ± 7	79 ± 6	78 ± 6
Mean 25-hydroxyvitamin D levels (nmol/L)	35 ± 7	62 ± 6 ^a^	112 ± 19 ^a,b^
Mean daily vitamin D intake in pregnancy (µg)	11.6 ± 3.5	17.5 ± 4.6 ^a^	40.0 ± 12.0 ^a,b^
Cumulative vitamin D intake in pregnancy (mg)	4.0 ± 1.4	5.8 ± 1.9 ^a^	12.9 ± 3.5 ^a,b^

Unless otherwise stated, the data are presented as the mean ± standard deviation. Body mass index and blood pressure represent mean values from visits during which 25-hydroxyvitamin D levels were registered. Group 1: sons of women with vitamin D deficiency; Group 2: sons of women with vitamin D insufficiency; and Group 3: sons of women with normal vitamin D status. ^a^ *p* < 0.05 vs. respective value in group 1; ^b^ *p* < 0.05 vs. respective value in group 2.

**Table 3:** Baseline characteristics of boys participating in the study.

Variable	Group 1	Group 2	Group 3
Number (n)	30	29	29
Gestational age of delivery (weeks)	39 ± 2	40 ± 2	40 ± 2
Birth order: first/second/third and subsequent (%)	50/40/10	52/42/7	48/42/10
Length (cm)	54.3 ± 1.6	54.1 ± 1.8	54.6 ± 1.5
Weight (kg)	4.47 ± 0.50	4.39 ± 0.52	4.55 ± 0.47
Body mass index (kg/m^2^)	15.2 ± 1.0	15.0 ± 1.1	15.3 ± 0.8
Head circumference (cm)	37.3 ± 0.6	37.4 ± 0.6	37.2 ± 0.7
Breastfeeding (%)	70	72	69
Total daily vitamin D intake (µg)	12.8 ± 1.4	13.0 ± 1.6	13.2 ± 1.7

Unless otherwise stated, the data are presented as the mean ± standard deviation. Group 1: sons of women with vitamin D deficiency; Group 2: sons of women with vitamin D insufficiency; and Group 3: sons of women with normal vitamin D status.

**Table 4:** Salivary testosterone concentrations in sons of women with different vitamin D statuses in pregnancy.

Age	Group 1	Group 2	Group 3
Month 1	220 ± 46	130 ± 38 ^f^	123 ± 37 ^f^
Month 2	182 ± 46 ^a^	155 ± 41 ^a,f^	149 ± 48 ^a,f^
Month 3	150 ± 50 ^a,b^	128 ± 39 ^b,f^	122 ± 43 ^b,f^
Month 4	118 ± 53 ^a,b,c^	90 ± 34 ^a,b,c,f^	84 ± 40 ^a,b,c,f^
Month 5	73 ± 49 ^a,b,c,d^	46 ± 22 ^a,b,c,d,f^	39 ± 18 ^a,b,c,d,f^
Month 6	42 ± 23 ^a,b,c,d,e^	35 ± 20 ^a,b,c,d,e^	32 ± 16 ^a,b,c,d,e^
Month 8	Below LOD	Below LOD	Below LOD
Month 10	Below LOD	Below LOD	Below LOD
Month 12	Below LOD	Below LOD	Below LOD

The data are expressed in pmol/L and presented as the mean ± standard deviation. Group 1: sons of women with vitamin D deficiency; Group 2: sons of women with vitamin D insufficiency; and Group 3: sons of women with normal vitamin D status. ^a^ *p* < 0.05 vs. concentrations at month 1 in the same study group; ^b^ *p* < 0.05 vs. concentrations at month 2 in the same study group; ^c^ *p* < 0.05 vs. concentrations at month 3 in the same study group; ^d^ *p* < 0.05 vs. concentrations at month 4 in the same study group; ^e^ *p* < 0.05 vs. concentrations at month 5 in the same study group; and ^f^ *p* < 0.05 vs. concentrations in group 1 at the same time point. Abbreviation: LOD—limit of detection.

**Table 5:** Salivary androstenedione concentrations in sons of women with different vitamin D statuses in pregnancy.

Age	Group 1	Group 2	Group 3
Month 1	96 ± 43	100 ± 55	104 ± 60
Month 2	102 ± 55	90 ± 48	110 ± 49
Month 3	108 ± 50	96 ± 42	118 ± 59
Month 4	90 ± 50	110 ± 51	102 ± 42
Month 5	62 ± 25 ^a,b,c,d^	70 ± 32 ^a,b,c,d^	65 ± 31 ^a,b,c,d^
Month 6	42 ± 21 ^a,b,c,d,e^	40 ± 20 ^a,b,c,d,e^	32 ± 18 ^a,b,c,d,e^
Month 8	Below LOD	Below LOD	Below LOD
Month 10	Below LOD	Below LOD	Below LOD
Month 12	Below LOD	Below LOD	Below LOD

The data are expressed in pmol/L and presented as the mean ± standard deviation. Group 1: sons of women with vitamin D deficiency; Group 2: sons of women with vitamin D insufficiency; and Group 3: sons of women with normal vitamin D status. ^a^ *p* < 0.05 vs. concentrations at month 1 in the same study group; ^b^ *p* < 0.05 vs. concentrations at month 2 in the same study group; ^c^ *p* < 0.05 vs. concentrations at month 3 in the same study group; ^d^ *p* < 0.05 vs. concentrations at month 4 in the same study group; and ^e^ *p* < 0.05 vs. concentrations at month 5 in the same study group. Abbreviation: LOD—limit of detection.

**Table 6:** Salivary DHEA-S concentrations in sons of women with different vitamin D statuses in pregnancy.

Age	Group 1	Group 2	Group 3
Month 1	156 ± 59	150 ± 61	146 ± 53
Month 2	140 ± 71	145 ± 70	141 ± 57
Month 3	154 ± 65	138 ± 60	158 ± 65
Month 4	142 ± 50	146 ± 55	138 ± 51
Month 5	159 ± 61	151 ± 56	142 ± 55
Month 6	162 ± 76	142 ± 59	147 ± 47
Month 8	143 ± 56	135 ± 62	140 ± 60
Month 10	152 ± 58	150 ± 70	138 ± 55
Month 12	157 ± 55	141 ± 51	152 ± 62

The data are expressed in nmol/L and presented as the mean ± standard deviation. Group 1: sons of women with vitamin D deficiency; Group 2: sons of women with vitamin D insufficiency; and Group 3: sons of women with normal vitamin D status. Abbreviations: DHEA-S—dehydroepiandrosterone sulfate.

**Table 7:** Salivary estradiol concentrations in sons of women with different vitamin D statuses in pregnancy.

Age	Group 1	Group 2	Group 3
Month 1	18 ± 10	16 ± 10	15 ± 10
Month 2	15 ± 8	16 ± 9	12 ± 7
Month 3	13 ± 7	11 ± 6	11 ± 6
Month 4	Below LOD	Below LOD	Below LOD
Month 5	Below LOD	Below LOD	Below LOD
Month 6	Below LOD	Below LOD	Below LOD
Month 8	Below LOD	Below LOD	Below LOD
Month 10	Below LOD	Below LOD	Below LOD
Month 12	Below LOD	Below LOD	Below LOD

The data are expressed in pmol/L and presented as the mean ± standard deviation. Group 1: sons of women with vitamin D deficiency; Group 2: sons of women with vitamin D insufficiency; and Group 3: sons of women with normal vitamin D status. Abbreviation: LOD—limit of detection.

**Table 8:** Salivary progesterone concentrations in sons of women with different vitamin D statuses in pregnancy.

Age	Group 1	Group 2	Group 3
Month 1	130 ± 68	115 ± 79	138 ± 65
Month 2	120 ± 79	130 ± 60	127 ± 74
Month 3	130 ± 65	118 ± 59	122 ± 57
Month 4	118 ± 67	112 ± 55	140 ± 70
Month 5	128 ± 70	127 ± 61	145 ± 78
Month 6	122 ± 70	120 ± 86	118 ± 62
Month 8	136 ± 34	140 ± 75	144 ± 64
Month 10	146 ± 75	123 ± 56	153 ± 80
Month 12	126 ± 55	126 ± 64	143 ± 78

The data are expressed in pmol/L and presented as the mean ± standard deviation. Group 1: sons of women with vitamin D deficiency; Group 2: sons of women with vitamin D insufficiency; and Group 3: sons of women with normal vitamin D status.

**Table 9:** Salivary 17-hydroxyprogesterone concentrations in sons of women with different vitamin D statuses in pregnancy.

Age	Group 1	Group 2	Group 3
Month 1	101 ± 43	95 ± 44	90 ± 48
Month 2	93 ± 47	92 ± 40	108 ± 66
Month 3	98 ± 40	104 ± 42	101 ± 52
Month 4	87 ± 40	102 ± 55	95 ± 40
Month 5	104 ± 51	112 ± 59	93 ± 37
Month 6	106 ± 58	93 ± 42	102 ± 48
Month 8	98 ± 43	88 ± 46	95 ± 40
Month 10	92 ± 31	96 ± 38	88 ± 42
Month 12	103 ± 42	90 ± 35	96 ± 50

The data are expressed in pmol/L and presented as the mean ± standard deviation. Group 1: sons of women with vitamin D deficiency; Group 2: sons of women with vitamin D insufficiency; and Group 3: sons of women with normal vitamin D status.

**Table 10:** Urinary FSH concentrations in sons of women with different vitamin D statuses in pregnancy.

Age	Group 1	Group 2	Group 3
Month 1	1.25 ± 0.35	0.90 ± 0.25 ^g,h^	0.75 ± 0.20 ^g^
Month 2	1.18 ± 0.30	0.88 ± 0.30 ^g,h^	0.68 ± 0.34 ^g^
Month 3	1.05 ± 0.28	0.85 ± 0.26 ^g,h^	0.65 ± 0.22 ^g^
Month 4	1.18 ± 0.40	0.92 ± 0.32 ^g,h^	0.71 ± 0.26 ^g^
Month 5	0.95 ± 0.25 ^a,b,c,d^	0.70 ± 0.25 ^a,b,c,d,g,h^	0.52 ± 0.20 ^a,b,c,d,g^
Month 6	0.55 ± 0.20 ^a,b,c,d,e^	0.38 ± 0.21 ^a,b,c,d,e,g^	0.40 ± 0.24 ^a,b,c,d,e,g^
Month 8	0.30 ± 0.15 ^a,b,c,d,e,f^	0.27 ± 0.18 ^a,b,c,d,e,f^	0.28 ± 0.16 ^a,b,c,d,e,f^
Month 10	Below LOD	Below LOD	Below LOD
Month 12	Below LOD	Below LOD	Below LOD

The data are expressed in international units per µmol of creatinine, and presented as the mean ± standard deviation. Group 1: sons of women with vitamin D deficiency; Group 2: sons of women with vitamin D insufficiency; and Group 3: sons of women with normal vitamin D status. ^a^ *p* < 0.05 vs. concentrations at month 1 in the same study group; ^b^ *p* < 0.05 vs. concentrations at month 2 in the same study group; ^c^ *p* < 0.05 vs. concentrations at month 3 in the same study group; ^d^ *p* < 0.05 vs. concentrations at month 4 in the same study group; ^e^ *p* < 0.05. vs. concentrations at month 5 in the same study group; ^f^ *p* < 0.05 vs. concentrations at month 6 in the same study group; ^g^ *p* < 0.05 vs. concentrations in group 1 at the same time point; and ^h^ *p* < 0.05 vs. concentrations in group 3 at the same time point. Abbreviations: FSH—follicle-stimulating hormone; and LOD—limit of detection.

**Table 11:** Urinary LH concentrations in sons of women with different vitamin D statuses in pregnancy.

Age	Group 1	Group 2	Group 3
Month 1	2.40 ± 0.50	1.55 ± 0.60 ^f^	1.50 ± 0.57 ^f^
Month 2	2.18 ± 0.46 ^a^	1.92 ± 0.52 ^a,f^	1.80 ± 0.51 ^a,f^
Month 3	1.76 ± 0.56 ^a,b^	1.50 ± 0.62 ^b,f^	1.51 ± 0.48 ^b,f^
Month 4	1.46 ± 0.47 ^a,b,c^	1.15 ± 0.42 ^a,b,c,f^	1.10 ± 0.39 ^a,b,c,f^
Month 5	1.15 ± 0.41 ^a,b,c,d^	0.85 ± 0.32 ^a,b,c,d,f^	0.80 ± 0.40 ^a,b,c,d,f^
Month 6	0.62 ± 0.38 ^a,b,c,d,e^	0.40 ± 0.28 ^a,b,c,d,e,f^	0.38 ± 0.20 ^a,b,c,d,e,f^
Month 8	Below LOD	Below LOD	Below LOD
Month 10	Below LOD	Below LOD	Below LOD
Month 12	Below LOD	Below LOD	Below LOD

The data are expressed in international units per µmol of creatinine, and presented as the mean ± standard deviation. Group 1: sons of women with vitamin D deficiency; Group 2: sons of women with vitamin D insufficiency; and Group 3: sons of women with normal vitamin D status. ^a^ *p* < 0.05 vs. concentrations at month 1 in the same study group; ^b^ *p* < 0.05 vs. concentrations at month 2 in the same study group; ^c^ *p* < 0.05 vs. concentrations at month 3 in the same study group; ^d^ *p* < 0.05 vs. concentrations at month 4 in the same study group; ^e^ *p* < 0.05. vs. concentrations at month 5 in the same study group; and ^f^ *p* < 0.05 vs. concentrations in group 1 at the same time point. Abbreviations: LH—luteinizing hormone; and LOD—limit of detection.

**Table 12:** Penile length in sons of women with different vitamin D statuses in pregnancy.

Age	Group 1	Group 2	Group 3
Month 1	3.5 ± 0.4	3.4 ± 0.4	3.5 ± 0.4
Month 2	3.7 ± 0.4	3.5 ± 0.3	3.6 ± 0.5
Month 3	3.8 ± 0.5 ^a^	3.5 ± 0.4	3.6 ± 0.4
Month 4	4.0 ± 0.4 ^a,b^	3.7 ± 0.4 ^a,b,f^	3.7 ± 0.4 ^a,f^
Month 5	4.2 ± 0.3 ^a,b,c^	3.9 ± 0.4 ^a,b,c,f^	3.9 ± 0.4 ^a,b,c,f^
Month 6	4.3 ± 0.4 ^a,b,c,d^	4.0 ± 0.3 ^a,b,c,d,f^	3.9 ± 0.4 ^a,b,c,f^
Month 8	4.3 ± 0.4 ^a,b,c,d^	4.0 ± 0.4 ^a,b,c,d,f^	4.0. ± 0.3 ^a,b,c,d,f^
Month 10	4.4 ± 0.5 ^a,b,c,d^	4.1 ± −0.4 ^a,b,c,d,f^	4.0 ± 0.4 ^a,b,c,d,f^
Month 12	4.4 ± 0.4 ^a,b,c,d^	4.2 ± 0.3 ^a,b,c,d,e,f^	4.1 ± 0.4 ^a,b,c,d,f^

The data are expressed in cm and presented as the mean ± standard deviation. Group 1: sons of women with vitamin D deficiency; Group 2: sons of women with vitamin D insufficiency; and Group 3: sons of women with normal vitamin D status. ^a^ *p* < 0.05 vs. value at month 1 in the same study group; ^b^ *p* < 0.05 vs. value at month 2 in the same study group; ^c^ *p* < 0.05 vs. value at month 3 in the same study group; ^d^ *p* < 0.05 vs. value at month 4 in the same study group; ^e^ *p* < 0.05 vs. value at month 5 in the same study group; and ^f^ *p* < 0.05 vs. value in group 1 at the same time point.

**Table 13:** Testicular volume in in sons of women with different vitamin D statuses in pregnancy.

Age	Group 1	Group 2	Group 3
Month 1	0.32 ± 0.07	0.32 ± 0.07	0.31 ± 0.05
Month 2	0.35 ± 0.06	0.34 ± 0.07	0.32 ± 0.06
Month 3	0.39 ± 0.06 ^a,b^	0.36 ± 0.05 ^a,e^	0.35 ± 0.06 ^a,b,e^
Month 4	0.42 ± 0.05 ^a,b,c^	0.39 ± 0.06 ^a,b,c,e^	0.37 ± 0.06 ^a,b,e^
Month 5	0.45 ± 0.06 ^a,b,c,d^	0.42 ± 0.05 ^a,b,c,d,e,f^	0.39 ± 0.05 ^a,b,c,e^
Month 6	0.46 ± 0.06 ^a,b,c,d^	0.43 ± 0.06 ^a,b,c,d,e,f^	0.40 ± 05 ^a,b,c,d,e^
Month 8	0.46 ± 0.07^.a,b,c,d^	0.43 ± 0.07 ^a,b,c,d,e,f^	0.40 ± 0.05 ^a,b,c,d,e^
Month 10	0.45 ± 0.06 ^a,b,c,d^	0.42 ± 0.06 ^a,b,c,d,e,f^	0.39 ± 0.05 ^a,b,c,e^
Month 12	0.45 ± 0.07 ^a,b,c^	0.42 ± 0.05 ^a,b,c,d,e,f^	0.39 ± 0.06 ^a,b,c,e^

The data are expressed in mL and presented as the mean ± standard deviation. Group 1: sons of women with vitamin D deficiency; Group 2: sons of women with vitamin D insufficiency; and Group 3: sons of women with normal vitamin D status. ^a^ *p* < 0.05 vs. value at month 1 in the same study group; ^b^ *p* < 0.05 vs. value at month 2 in the same study group; ^c^ *p* < 0.05 vs. value at month 3 in the same study group; ^d^ *p* < 0.05 vs. value at month 4 in the same study group; ^e^ *p* < 0.05 vs. value in group 1 at the same time point; and ^f^ *p* < 0.05 vs. value in group 3 at the same time point.

**Table 14:** Linear regression analysis of associations between mean gestational 25-hydroxyvitamin D levels and indices of male minipuberty during the first six months of life.

Variable	Month 1	Month 2	Month 3	Month 4	Month 5	Month 6
Testosterone	−0.352/0.269 *	−0.342/0.255 *	−0.320/0.245 *	−0.305/0.214 *	−0.280/0.188 *	−0.265/0.162 *
FSH	−0.467/0.255	−0.488/0.315 *	−0.462/0.288 *	−0.437/0.273 *	−0.412/0.282 *	−0.395/0.242 *
LH	−0.351/0.226 *	−0.348/0.224 *	−0.295/0.208 *	−0.284/0.196 *	−0.290/0.186 *	−0.275/0.157 *
Penile length	−0.034/0.055	−0.040/0.062	−0.082/0.151 *	−0.075/0.162 *	−0.100/0.180 *	−0.088/0.170 *
Testicular volume	−0.046/0.037	−0.048/0.042	−0.104/0.148 *	−0.112/0.174 *	−0.121/0.176 *	−0.108/0.158 *

The first value denotes the beta coefficient of the regression model, while the second value denotes the coefficient of determination. Statistically significant results (*p* < 0.05) are marked with an asterisk.

## Data Availability

The data that support the findings of this study are available from the corresponding author upon reasonable request.

## Abbreviations

DHEA-S	dehydroepiandrosterone sulfate
FSH	follicle-stimulating hormone
LH	luteinizing hormone
LOD	limit of detection
